# Time Delay and Frequency Analysis of Remote Microphones

**DOI:** 10.3390/audiolres15050123

**Published:** 2025-09-25

**Authors:** Elena Andreatta, Igor Caregnato, Antonio Selmo, Andrea Gulli, Marius George Onofrei, Eva Orzan

**Affiliations:** 1 Acustica Caregnato, Marostica, 36063 Vicenza, Italy; elena.andreatta77@gmail.com (E.A.); igor.caregnato@gmail.com (I.C.); 2 Independent Researcher, Albaredo d’Adige, 37041 Verona, Italy; selmo.ing@tin.it; 3 Department of Management and Engineering, University of Padova, 36100 Vicenza, Italy; 4 Human-Computer Interaction Lab, University of Udine, 33100 Udine, Italy; onofrei.mariusgeorge@spes.uniud.it; 5 Institute for Maternal and Child Health—IRCCS Burlo Garofolo, 34137 Trieste, Italy; eva.orzan@burlo.trieste.it

**Keywords:** remote microphone, hearing aid, transparency, electroacoustic measurements, time delay, frequency response, tolerable time delay

## Abstract

**Background/Objectives:** A.BA.CO. is a speech-to-text captioning system developed for school classrooms. The system uses remote microphones to capture the teacher’s speech without background noise. Under this setup, an issue of signal latency arises for students wearing hearing aids (HAs) or cochlear implants (CIs), whose latency is different from that of the remote microphones and may require the development of a temporal coupling solution. This study establishes the foundation for such a solution by determining the latency of two RMs (Remote Microphones) compatible with both HA and CI systems. The frequency response of the systems is analyzed independently and combined. **Methods:** The RMs combined with two Behind-The-Ear HAs, for which transparency was verified, were tested with two different compression ratios in a laboratory specializing in electroacoustic measurements using the comparison method to assess performance. **Results:** The time measurements revealed that the RMs differ by 10–12 ms (23–24 ms and 33–35 ms) and that the two HAs have time delays that differ by 1–2 ms (6–7 ms and 5–7 ms). The frequency responses showed that when HA and RM have similar gains, they exhibit comb-filter distortions. This effect could alter the acoustic output of devices in the ear canal and vary according to the mix ratio and mutual positions of HA and RM, potentially necessitating greater commitment from the wearer. **Conclusions:** The communication system will have to foresee different delays based on the model and brand of RM because similar transmission systems do not have the same time delays. RMs were originally designed for HA and are most effective if they represent the only or major acoustic stimulation that reaches the eardrum. These limits must be considered when estimating the effectiveness of A.BA.CO. with RM.

## 1. Introduction

Research indicates that children need constant and complete access to acoustic and verbal stimulation to develop their communication, linguistic, cognitive, and psychosocial skills. The school is a stimulating environment, but it has many acoustic aspects that create difficulties for children with hearing disabilities. Several studies have investigated these critical issues by analyzing the acoustics of school classrooms [1,2]. In school buildings, background noise levels can easily exceed the maximum levels of 35 dB (A) established by the World Health Organization [3]. These noise levels strongly mask speech. The American Speech-Language-Hearing Association [4] recommends a signal-to-noise ratio (SNR) greater than 15 dB for students with normal hearing. The American National Standards Institute and the Acoustical Society of America [3] indicate reverberation times of 0.6 s for students with normal hearing and 0.3 s for hearing-impaired students with both hearing aids (HAs) [2] and cochlear implants (CIs) [5]. Low signal-to-noise ratio affects younger children more, while the effects of reverberation do not appear to vary with age [6].

Improving the acoustics of classrooms that do not meet the reference standards is essential. However, it is also necessary to take into account the student’s abilities, such as the characteristics of the hearing problem, the threshold amplified by different hearing aids, the early or late ability to communicate in two languages, visuospatial working memory skills, auditory and linguistic attention [7], and any other disabilities [8]. The distance from the speaker, the speaker’s oratory characteristics, such as accent and gestures, and the familiarity with amplification devices are key factors [9,10]. These factors, together with the number of students in the class and the complexity of the tasks, significantly influence the student’s ability to recognize and understand speech [11].

Although hearing aids improve the audibility of the speech signal, they cannot remove the obstacles to speech understanding; thus, the use of remote microphones (RM) is recommended, especially during school activities. They collect the primary signal near the source and transmit it to the assistive device, avoiding degradation due to background noise, reverberation, or distance. The operation of the RM includes a transmission and a reception phase, and the different technologies differ depending on the transmission methods. The most developed and used technologies are:

- Electromagnetic induction captured by the hearing aid telecoil;
- Near-field magnetic induction integrated with the Bluetooth standard;
- Frequency modulation with a constant demodulated carrier frequency;
- Digital radio frequency, providing bidirectional exchanges via frequency hopping spread spectrum (FHSS).

RM can improve SNR by up to 20 dB. The benefit decreases by 3 dB when used only in one ear [12]. It can vary from 4 to 5 dB depending on the mix ratio, setting directionality, and adaptive gain. In practice, the SNR decreases when the activity of the HA microphones paired to the RM increases [10,13,14].

According to Schafer’s literature review [15], the improvements due to RM combined with HA and CI are greater with digital adaptive systems and bilateral or bimodal configurations. The use of RM is also favorable for children suffering from dyslexia, ADHD, speech disorders, and auditory processing. There is documented evidence of positive effects for preschool children [16] and children with ASD [17].

Speech recognition in the presence of masking noise improves with development [18]; however, the neural mechanisms underlying this ability remain plastic and sensitive to experience until adolescence [19]. This highlights the continued importance of optimizing listening conditions.

Today’s technology offers combined acoustic and visual aids that support daily life. These tools can be integrated into schools to strengthen inclusion and help children develop cognitive and socio-emotional skills. A.BA.CO. (Abbattimento delle BArriere di COmunicazione) focuses on technology and services for inclusion, education, and accessibility for hearing-impaired individuals. Its goal is to create a system that enhances educational inclusion, starting with improved oral communication. The system aims to foster communicative autonomy and support participation in school life. It will convert the teacher’s voice into accurate subtitles and deliver a high-quality, clear acoustic signal to the student’s hearing devices.

This study assumes that speech synthesis systems require different delay times than hearing aids. Audiological standards do not cover these delay times, and manufacturers’ data sheets usually do not report them. The use of a remote microphone (RM) combined with a hearing aid (HA) introduces latency caused by signal transmission, digital conversion, and processing. The following analysis focuses on measuring this latency. The method used is precise, reliable, and innovative. We measured the delay in RM and HA systems with high sensitivity and over a sufficiently long time range. Current commercial instruments do not offer this capability. This study hypothesizes that commercially available RM systems differ in latency due to proprietary transmission protocols, and that these differences, when combined with HA latency, can result in comb filtering effects impacting sound quality.

Scientific literature widely confirms the effectiveness of remote microphones (RM) in many contexts. Recent research highlights the increased risk of listening effort and fatigue in children with hearing loss. Improving audibility alone does not always reduce listening effort in noisy environments. Cognitive load and motivation also play a key role [20]. When listening requires sustained effort beyond the child’s available or willing cognitive resources, it can lead to fatigue and exhaustion [21]. Hornsby and colleagues recently examined listening-related fatigue in children and its consequences. Their research, despite the absence of a specific measurement scale, confirms that children with hearing loss are at high risk of experiencing fatigue, regardless of the type or severity of the loss. This fatigue can cause physical symptoms (tiredness, headaches, and the need to remove hearing aids or take a nap), cognitive problems (reduced concentration, drops in attention, and disengagement), and socio-emotional effects (sadness, irritability, and withdrawal from social situations). Children often fail to recognize the cause of their discomfort, and there is no standardized intervention protocol. The authors recommend that professionals assess listening-related fatigue carefully and adopt a multidisciplinary approach to address it. Real-world experiments show that the RM benefit is highly dependent on signal delay and environmental conditions, which vary with microphone distance and system architecture [22].

Building on these premises, the study measures the RM’s time delay and evaluates its electroacoustic performance. This assessment supports the design of the A.BA.CO. system and the analysis of the prototype’s experimental results.

## 2. Materials and Methods

Two RM models were selected for time delay and frequency analysis: the Phonak Roger Select (Sonova, Stäfa, Switzerland) and the ReSound Multi Mic (GN Group, Ballerup, Denmark). Both transmit digital radio signals to hearing aids (HA) and cochlear implants (CI). These devices convert audio into binary digital signals and transmit them via ultra-high-frequency carrier waves at 2.4 GHz, using bidirectional communication. They operate on closed transmission platforms developed by the manufacturers, within the same frequency band as standard Bluetooth but with lower latency. Both use Frequency Hopping Spread Spectrum (FHSS), which rapidly switches carrier frequencies in a random but synchronized pattern, reducing interference and power consumption while ensuring a stable, uninterrupted connection [23]. The two HA/RM pairs were selected because they were among the few commercial systems that explicitly reported latency values in their technical datasheets, with delays within a tolerable range for synchronous speech-to-text applications such as A.BA.CO.

During the measurements, the RM was linked to HA Behind-The-Ear (BTE), which is mostly used in pediatric applications with a custom-made unventilated ear mold. The Phonak Sky M90 SP and ReSound Enzo Q988 DWHT are digital BTEs of similar power, technical specifications, and electro-acoustic characteristics, with 20 and 17 channels, respectively. The specifications are displayed in Table 1.

To reduce the effect of proprietary algorithms, the HA settings disabled directionality, noise reduction, feedback cancelation, and expansion. Two configurations with different compression ratios were tested, both based on the DSL v5 pediatric formula. The first setting (FIT1) used a linear compression ratio (1:1). It simulated a flat hearing loss from 125 Hz to 8 kHz, with thresholds at 55 dB HL and UCL at 105 dB HL. Measurements were made with TDH39P supra-aural headphones (Telephonics, Huntington, NY, USA). The second setting (FIT2) used an average compression ratio of 2.2:1 (HA1) and 2.1:1 (HA2). It simulated a more severe hearing loss, with thresholds at 80 dB HL and UCL at 115 dB HL. Electroacoustic tests were performed with an Otometrics Aurical HIT (GN Otometrics, Taastrup, Denmark), using both standard and FreeStyle modes. Calibration from March 2021 and weekly self-checks ensured reliable measurements [23]. Gain values at 500, 800, 1000, 1600, 2000, and 2500 Hz are shown in Table 2 for comparison with lab data.

HA and RM were set so that their microphones provided the same amplification. The RM should not alter the HA’s frequency response. This condition, called transparency, was verified following the American Academy of Audiology (AAA) guidelines [26]. The test used an ISTS signal at 65 dB SPL for 25 s in a 2cc coupler with an HA2 adapter. Measurements were taken in three steps: first, the HA alone in the test box; second, the HA in the test box paired with the RM (with the HA microphones muted); and third, the RM in the test box in omnidirectional mode, connected to the HA placed outside the box. The average gain differences at 750, 1000, and 2000 Hz stayed within the ±2 dB tolerance, as shown in Table 3. The table also reports data for 500, 800, 1600, and 2500 Hz. To achieve transparency, adjustments were needed: the HA1 volume was reduced by two steps, and the RM2 volume was increased by two steps. Transparency for CI connected to RM can be verified using the same AAA protocol [27]. However, it may be harder to achieve in bimodal fittings since most RMs are preset to match HA gain levels [28].

**Figure 1:**
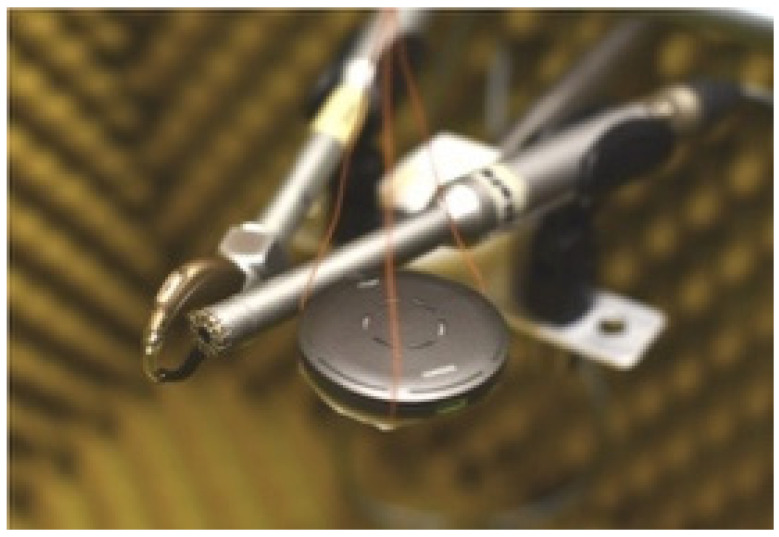
Close positioning of the HA on a 2cc coupler with measurement microphone, reference microphone, and RM.

The measurements aimed at characterizing the behavior of two RM in the time and frequency domain were carried out in a laboratory specialized in electroacoustic measurements with a semi-anechoic chamber. Inside the semi-anechoic chamber (80 × 150 × 80 cm), the acoustic stimulations generate stationary waves with resonance frequencies around 90 Hz, not interfering with the measurement range of HA, defined between 100 and 125 Hz and 8–10 kHz. The laboratory is far from sources of continuous noise that reach a maximum of 50 dB (A), and the characterization measurements of the semi-anechoic chamber have detected an attenuation capacity of environmental noise of 30 dB in the upper part of the spectrum, and of 20 dB in the lower part. In the semi-anechoic chamber, a 6” cone speaker for low frequencies (100–1250 Hz) and a 2” dome speaker for high frequencies (1–10 kHz) are almost superimposed with acoustic centers on the same vertical axis; therefore, in addition to covering the entire required frequency range, they avoid the interference of phase differences generated by two sources distant from each other. Two half-inch microphones with frequency response from 20 Hz to 20 kHz are installed a few millimeters apart and at the same distance of 115 cm from the speaker and two-way (Figure 1).

Time and frequency domain measurements used a comparison method. The reference microphone, calibrated for audiometric use, recorded the input signal to verify the accuracy of the stimulation. This signal was compared to the output recorded by the measurement microphone placed in the 2cc coupler connected to the HA. To keep the acoustic stimulation consistent, the RM was placed as close as possible to the reference microphone. Both were positioned 115 cm from the two-way speaker (Figure 1).

This setup is based on the idea that verifying RM transparency using the AAA or the European Union of Hearing Aid Acousticians (EUHA) [29] protocols does not fully reflect real-life use. In real conditions, the test signal should reach both the HA and RM simultaneously [28]. However, this is not possible to reproduce accurately. In real use, the RM is placed closer to the sound source, while the HA is farther away. This setup could not be replicated due to the limits of the test box and the need to follow the comparison method. The electroacoustic verification procedures adopted in this study align with standardized RMS transparency guidelines [30].

The measurement setup has two parts: stimulation and measurement. Using the reference microphone and comparing the input signal with the device output ensures accurate gain measurement. The microphone amplification chain (reference and measurement in the 2cc coupler) has a frequency response from 20 Hz to 50 kHz, with a tolerance of ±1 dB.

The instrumentation includes three Tektronix SG505 (Tektronix, Shanghai, China) oscillators, a Rhode & Schwarz SPN oscillator (Rhode & Schwarz, Munich, Germany), a Hewlett-Packard HP 651B test oscillator (Hewlett Packard, Palo Alto, CA, USA), a Hewlett-Packard 3325B function generator, a Siglent SDG1010 function generator (Siglent Technologies, Shenzen, China), and an UnaOhm EM139 function generator (UnaOhm, Brignano Gera d’Adda, Italy). Measurement devices include a Hewlett-Packard 5335A counter, a Hameg HM8031-3 frequency meter (HAMEG Instruments, Mainhausen, Germany), two multimeters (HP3456A and HP34401A), a Brüel & Kjær B&K2632 measurement amplifier (Brüel & Kjær, Nærum, Denmark), and two spectrum analyzers (HP3580A and HP3585A with linear/logarithmic scale software). The setup also uses a Behringer Ultra-Graph PRO 31-band graphic equalizer (Behringer, Willich, Germany), two Behringer Ultra-Q PRO parametric equalizers, a Siemens D110 600-ohm attenuator (Siemens, Munich, Germany), a Behringer Ultra-Gain PRO microphone amplifier, and a Behringer Reference Amplifier A500. For acoustic measurement, two Behringer ECM 8000 ½” microphones are used, along with a Tektronix 7904 and a LeCroy 9354L oscilloscope (Teledyne LeCroy, Chestnut Ridge, New York, NY, USA). Before starting the measurements, the microphone preamplifiers were calibrated to ensure the output signals matched within a maximum error of 1 dB, up to 70–80 dB SPL. Since it was not possible to obtain a flat sinusoidal output from the speaker with constant electrical power, equalization was needed. A 31-band graphic equalizer (1/3 octave, 20 Hz to 20 kHz) and a 5-band parametric equalizer were used for each amplification channel feeding the two-way speaker.

**Figure 2:**
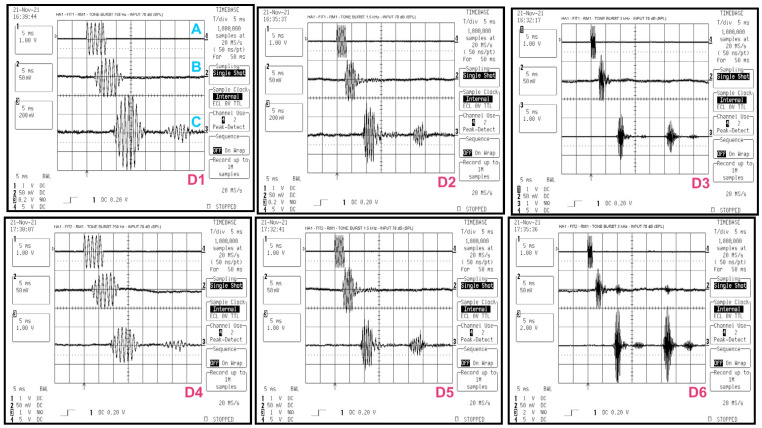
Time domain measurements graphs of HA1 and RM1, with Time/Div of 5 ms and time window of 50, in quadrants D1 and D4 with sinusoidal tone burst stimulus at 750 Hz, respectively, with FIT1 and FIT2 where A represents the electrical signal sent to the speaker, B the electrical signal coming from the reference microphone and C the electrical signal coming from the measurement microphone connected to the 2cc coupler. In quadrants D2 and D5 with sinusoidal toneburst stimulus at 1500 Hz, respectively, with FIT1 and FIT2, and in quadrants D3 and D6 with sinusoidal toneburst stimulus at 3000 Hz, respectively, with FIT1 and FIT2.

To measure time delay, tone bursts of 750, 1500, and 3000 Hz were generated. Each tone lasted for 5 cycles at 70 dB SPL. The oscilloscope displayed the time evolution of the signals. From this, the delay between the tone burst reaching the reference microphone and the measurement microphone in the 2cc coupler was measured. It also showed the delay between the electrical signal sent to the speaker and the acoustic signal captured by the reference microphone, as shown in quadrant D1 of Figure 2.

For the frequency response, a sinusoidal sweep was used. It covered two ranges: 100–1000 Hz and 1–10 kHz. The same microphone setup was used to test the frequency behavior of the HA alone, the HA combined with the RM (muted), and the HA combined with the active RM. Each frequency range was sampled with 512 points for high accuracy. The bi-amplification and equalization chain ensured the acoustic signal remained as constant as possible across the sweep.

Harmonic distortion was evaluated using a low-distortion oscillator with high spectral purity. However, this measurement was not performed because the signal-to-noise ratio (SNR) of about 40 dB would not yield meaningful results.

## 3. Results

### 3.1. Time Domain Responses

**Figure 3:**
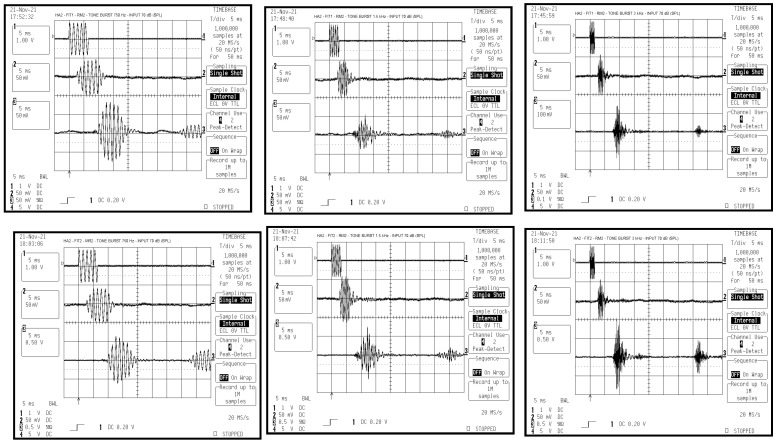
Graphs of the time domain measurements of HA2 and RM2, with Time/Div of 5 ms and time window of 50 ms, arranged as described in Figure 2.

Measurements with tone bursts at 750, 1500, and 3000 Hz (5 cycles at 70 dB SPL) showed that HA1 has a delay of about 6–7 ms from the arrival of the acoustic input for both FIT1 and FIT2. RM1 showed a total delay of about 23–24 ms, which includes both the transmission delay and the HA1 delay (Figure 2). HA2 has a delay between 5 and 7 ms, which decreases by approximately 2 ms as the tone frequency increases, for both FIT1 and FIT2. RM2 showed a total delay of 33–35 ms. The 2 ms variation with frequency appears linked to HA2, not the RM transmission protocol (Figure 3). During testing, some output responses showed non-deterministic behavior in reaction to steady sinusoidal signals. These responses were not suitable for accurate gain evaluation [30].

### 3.2. Frequency Responses in the Lower Part of the Spectrum

**Figure 4:**
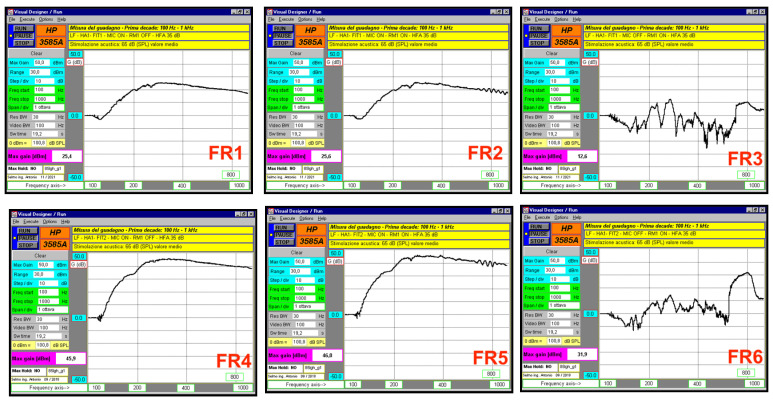
HA1 and RM1 responses with a frequency sweep from 100 Hz to 1 kHz at 65 dB SPL, where the three upper graphs represent the responses with FIT1 and the lower three with FIT2. Graphic FR1 represents the response with FIT1 of HA1 paired with RM1 mute, FR2 the response of HA1 coupled with RM1, and FR3 the response of HA1 muted coupled with RM1. Similarly, graph FR4 represents the response with FIT2 of HA1 paired with RM1 mute, FR5 the response of HA1 paired with RM1, and FR6 the response of silenced HA1 paired with RM1.

**Figure 5:**
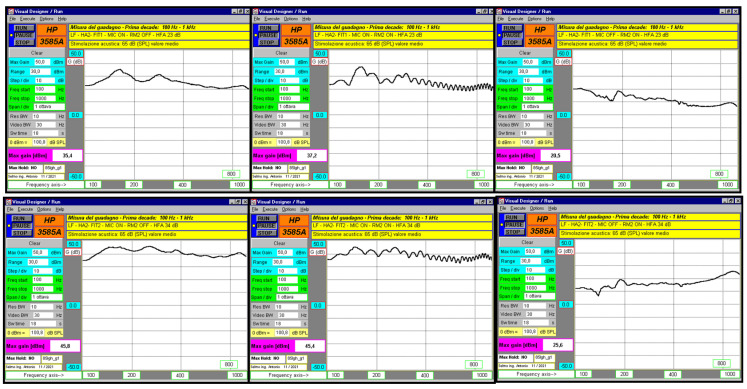
HA2 and RM2 responses with a frequency sweep from 100 Hz to 1 kHz at 65 dB SPL, with graphs distributed as described in Figure 4.

Frequency domain measurements used a sinusoidal sweep from 100 Hz to 1000 Hz at 65 dB SPL for both FIT1 and FIT2. Results showed that HA1 paired with RM1 (microphones on) and HA1 paired with RM1 (microphones off) had similar responses. In contrast, HA1, with its microphones off and paired with RM1, showed much lower gain across all frequencies (Figure 4, Table 4). The strikethrough line in the figure indicates when the device is disabled. For HA2, the setup with microphones off while paired with RM2 also showed reduced gain. HA2 paired with RM2 (microphones off) did not match the response of HA2 paired with RM2 (microphones on). In both FIT1 and FIT2, HA2 paired with RM2 showed distortions, likely caused by a comb filtering effect (Figure 5, Table 4).

### 3.3. Frequency Responses in the Upper Part of the Spectrum

Frequency domain measurements with a sinusoidal sweep from 1 kHz to 10 kHz at 65 dB SPL, for both FIT1 and FIT2, showed that both HAs paired with RM (microphones off) had slightly reduced gain. The responses of the HAs paired with RM (microphones off) did not match the responses when the RM was active with microphones on. In the latter case, gain could not be measured accurately due to fluctuations greater than ±2.5 dB. These variations were more noticeable with RM1, likely due to stronger comb filtering effects. While visual inspection of frequency response graphs suggests the presence of spectral distortion due to comb filtering, quantitative analysis of harmonic distortion and variance in gain deviation was not feasible due to the signal-to-noise limitations of the measuring setup, i.e., the instrument sensitivity and the HA’s background noise. This is acknowledged as a limitation. The corresponding gain values at 1600, 2000, and 2500 Hz are summarized in Table 5. Entries marked with “?” indicate cases where reliable values could not be obtained due to unstable high-frequency responses and measurement limitations.

## 4. Discussion

This study aimed to measure the time delay of two RMs commonly used in classrooms. The goal was to analyze how they temporally align with an innovative speech-to-text captioning system. Simultaneous measurements were taken with the HA and RM placed inside a semi-anechoic chamber, close to the reference microphone and each other (Figure 1). The stimulus was continuously monitored to ensure consistency. This high-precision method overcomes the limitations of the non-simultaneous measurements used in transparency verification protocols [28]. However, this setup does not reflect real-world use. In classrooms, the HA user is farther from the sound source and often in noisy environments. The RM improves the signal-to-noise ratio (SNR) only when placed very close to the speaker. It should be noted that our results, obtained in semi-anechoic conditions, represent an idealized scenario. In real classrooms, reverberation and background noise could amplify the perceptual impact of both delay and frequency response mismatches, potentially exacerbating comb filtering effects. These results are consistent with previous findings that suggest a tradeoff between clarity and externalization when remote microphones are directly injected into HA processing without spatial cues [31]. Nevertheless, while prior studies have simulated RM processing or evaluated isolated features, our study empirically compares commercial HA–RM combinations, highlighting brand-specific differences in time delay and frequency response not previously quantified in this way.

The measurements showed that RM1 has a time delay of 23–24 ms, while RM2 has a delay of 33–35 ms. This gap is due to the different digital transmission protocols used by the two manufacturers. RM2 also showed a variation in delay of more than 2 ms between tone bursts at 750 Hz and 3000 Hz. This delay is likely linked to the same behavior observed in HA2 and explained by filtering techniques that simulate cochlear critical bands. These filters have narrower passbands at low frequencies, causing processing times to vary slightly with frequency. Conversely, FFT-based techniques use constant amplitude bands and have fixed processing times [23]. The time behavior of the RM is influenced by the HA. However, functions that were disabled during testing—such as directionality, noise reduction, feedback cancelation, and expansion—have a minimal impact on time delay, less than 0.1 ms, though they significantly affect power consumption [22].

The time delay does not change between the two compression settings: FIT1 (1:1) and FIT2 (2.2:1 and 2.1:1). The RM shows a clear reduction in gain compared to the two HAs (see Figure 2 and Figure 3). Tone bursts with very short durations produce altered responses in the HA. As a result, the temporal response dynamics cannot be reliably evaluated [22,31]. The digital processing in HA and RM introduces delays that act like artificial echoes. This can cause comb filtering effects [32], which degrade speech quality when time misalignments exceed 10 ms [22]. Our findings that delay increases across RM brands and configurations are in agreement with previous work showing significant changes in RM behavior with increasing distance and TDOA [33].

When signals reach the ear with similar intensity but different delays, sound quality worsens, often leading to user discomfort [34]. These measurements do not reflect open-fitting conditions, where the ear also receives direct sound. In open fittings, the combined temporal and frequency responses of the HA, the RM, and the direct path alter perception. It is known that increasing the vent size reduces clarity and sound quality but increases the sensation of externalized sound [35].

**Figure 6:**
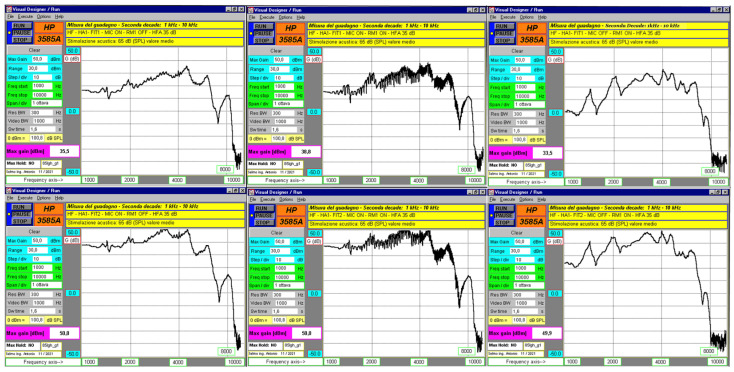
HA1 and RM1 responses with a frequency sweep from 1 kHz to 10 kHz at 65 dB SPL, with graphs distributed as described in Figure 4.

**Figure 7:**
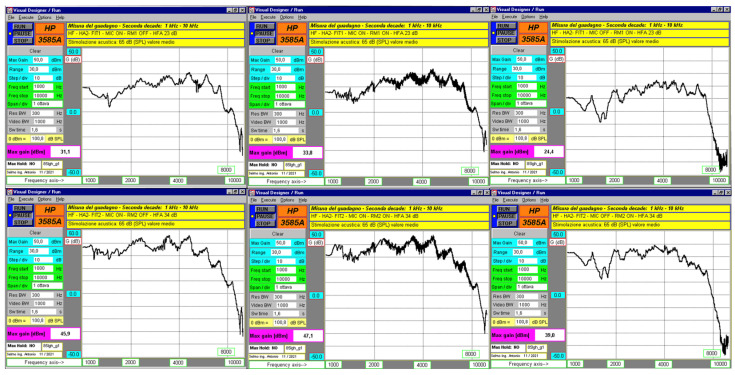
HA2 and RM2 responses with a frequency sweep from 1 Hz to 10 kHz at 65 dB SPL, with graphs distributed as described in Figure 4.

Temporal measurements were taken inside a semi-anechoic chamber with the devices placed close to each other and 115 cm from the speaker. The maximum time delay between HA and RM was about 16–17 ms for HA1–RM1 and about 27–28 ms for HA2–RM2. This delay changes depending on the relative positions of HA, RM, and the sound source. It can also affect the frequency response. Frequency domain analysis showed that when HA and RM have similar frequency sweep responses at 65 dB SPL, distortions become more noticeable. This happens due to a non-coherent vector sum, causing comb filtering artifacts in the speech frequency range [36]. In the higher frequency range, RM1 shows stronger distortions caused by comb filtering. This is likely because its delay relative to HA1 is lower than that of RM2 relative to HA2, and RM1 does not exhibit the same frequency-dependent delay seen in HA2–RM2 (Figure 6 and Figure 7). The frequency sweep results at 65 dB SPL using the Aurical HIT (Table 2) do not match the laboratory measurements. This difference may be due to the greater accuracy of the lab system, which collects 512 data points per frequency decade. The system also adjusts sweep duration by frequency range, 18 to 19.2 s for low frequencies and 1.6 s for high frequencies.

The values from the transparency check with the ISTS signal at 65 dB SPL for 25 s using the Aurical HIT are shown in Table 3. They match those in Table 2, except for high frequencies in the FIT2 settings of both HAs. These measures are based on frequency sweeps with pure tones at 65 dB SPL. RM transparency is confirmed by the average gain differences at 750, 1000, and 2000 Hz, as shown in Table 3. However, the laboratory’s simultaneous measurements of HA and RM produced very different results. These tests were performed with both devices placed close to each other and at the same distance from the speakers, using pure tone sweeps at 65 dB SPL with 512 points per frequency decade. Time delay is tolerable as long as it does not degrade sound quality. Studies by Stone and Moore show that delays over 10 ms reduce sound quality, with discomfort increasing in proportion to the severity of hearing loss. Hearing-impaired listeners tolerate longer delays than those with normal hearing—about 20–30 ms for mild to moderate losses and up to 40 ms for moderately severe losses. However, tolerance decreases when the delay changes with frequency or when visual cues are missing [34,37]. Goehring’s recent study confirms these findings [38], concluding that adults with hearing loss can generally tolerate time delays of 20–30 ms.

We show that devices achieving nominal transparency in gain can still present problematic delays, indicating that electroacoustic verification alone is insufficient without assessing temporal alignment. The temporal differences between acoustic signals can cause noticeable and unpleasant changes in sound quality. When signals arrive at the ear with different delays, they create artificial echoes that may reduce speech intelligibility. Issues with time delay and phase alignment can be hidden factors affecting whether patients feel satisfied with their hearing devices or not [32]. This study examined the time delay of two RMs, but further research is needed to understand the role of phase differences and to define acceptable time delay limits for school-age children.

## 5. Conclusions

The two RMs tested, although using the same transmission system and compatible with HA and CI, show time delays that differ by 10–12 ms. As a result, the A.BA.CO. system, combined with speech-to-text captions, will require different time adjustments depending on the RM used.

When the RM and HA have similar frequency response levels, comb filter distortions become more noticeable.

Studies show that hearing-impaired users can generally tolerate time delays of 20–30 ms. Only one of the two RMs stays within this range, even under the worst spatial conditions tested. This RM also shows stable time delays across frequencies. However, when set transparently, it produces stronger frequency distortions. To our knowledge, this is the first study to link empirical HA–RM timing mismatches with design considerations for multimodal captioning systems, opening new directions for pediatric hearing aid research.

To properly evaluate the effectiveness of the A.BA.CO. system, it is essential to understand the possibilities and limitations of hearing devices. This requires both subjective and objective tests across different settings, including combinations with CI and bimodal configurations, which were not explored in this study.

## Figures and Tables

**Table 1:** Technical specifications and electroacoustic characteristics of the selected RM and HA. * Measured with 2cc coupler according to [24,25].

RM	CI Connection	Mode of Transmission		Audio Frequency Range	MASL
Phonak Roger Select	Advanced Bionics	2.4 GHz ISM band		100 Hz–7.3 kHz	125 mA/m (with Roger NeckLoop)
ReSound Multi Mic	Cochlear	2.4 GHz		100 Hz–8 kHz	100 mA/m
HA *	**Audio Frequency Range**	**MASL**	**OSPL 90**	**FOG 50**	**Total Harmonic Distortion**
Phonak Sky M90 SP	100–5000 Hz	HFA 98 dB SPL	PEAK 139 dB SPL HFA 128 dB SPL	PEAK 81 dB SPL HFA 67 dB SPL	500 Hz 2.0%–800 Hz 1.0% 1.6 kHz 2.0%–3.2 kHz 1.0%
ReSound Enzo Q988 DWHT	100–4940 Hz	HFA 114 dB SPL	PEAK 134 dB SPL HFA 130 dB SPL	PEAK 73 dB SPL HFA 67 dB SPL	500 Hz 2.8%–800 Hz 0.4% 1.6 kHz 0.4%–3.2 kHz 0.1%

**Table 2:** HA Frequency Gain with pure tone frequency sweep at 50, 65, and 80 dB SPL.

Pure Tone Frequency Sweep	Fitting	HA Brand	Frequency Gain dB								
			500 Hz	750 Hz	800 Hz	1 kHz	1.6 kHz	2 kHz	2.5 kHz	HFA	PEAK
50 dB SPL	FIT1	HA1	23	21	21	19	19	25	28	22	39
		HA2	23	21	22	23	17	25	28	22	35
	FIT2	HA1	44	43	43	42	42	46	50	45	54
		HA2	40	40	40	42	38	45	46	42	49
65 dB SPL	FIT1	HA1	23	21	21	19	19	25	28	22	38
		HA2	23	21	22	23	17	25	28	23	35
	FIT2	HA1	39	35	35	34	32	36	40	35	44
		HA2	34	34	35	36	30	35	36	34	41
80 dB SPL	FIT1	HA1	22	18	18	17	17	20	25	20	35
		HA2	23	21	22	23	17	23	26	22	33
	FIT2	HA1	31	26	25	24	22	26	30	25	36
		HA2	26	26	27	28	19	27	27	25	33

**Table 3:** HA and RM frequency gain in AAA Transparency Verification protocol with speech-like ISTS stimulus performed at 65 dB SPL.

ISTS at 65 dB SPL Speech-Like Stimulus	Fitting	HA-RM Brand	Frequency Gain dB						
			500 Hz	750 Hz	800 Hz	1 kHz	1.6 kHz	2 kHz	2.5 kHz
STEP 1 Testing HA	FIT1	HA1	23	20	20	19	19	25	28
		HA2	24	21	22	23	17	24	27
	FIT2	HA1	39	36	37	37	38	45	50
		HA2	34	25	36	38	35	42	44
STEP 2 Testing HA connected to RM in mute modality	FIT1	HA1 + RM1 mute	23	20	20	19	19	25	28
		HA2 + RM2 mute	24	21	22	23	17	24	27
	FIT2	HA1 + RM1 mute	39	36	37	37	38	45	50
		HA2 + RM2 mute	34	25	36	38	35	43	44
STEP 3 Testing RM unmuted connected to HA	FIT1	RM1 + HA1	20	20	20	17	15	24	26
		RM2 + HA2	18	20	22	25	17	24	24
	FIT2	RM1 + HA1	36	36	38	36	36	49	50
		RM2 + HA2	30	25	36	40	35	43	40

**Table 4:** Gains at 500, 800, and 1000 Hz with a frequency sweep from 100 Hz to 1 kHz at 65 dB SPL in the various configurations.

Fit	Brand	HA + RM Output (Input 65 dB SPL)			HA + RM Output (Input 65 dB SPL)			HA + RM Output (Input 65 dB SPL)		
		Low Frequency Gain (dB)			Low Frequency Gain (dB)			Low Frequency Gain (dB)		
		500 Hz	800 Hz	1000 Hz	500 Hz	800 Hz	1000 Hz	500 Hz	800 Hz	1000 Hz
1	1	21.5	19.7	17.1	21.7	20.5	16.9	−10	8.4	4.8
	2	23	22.1	19.6	22 ± 2.5	22 ± 2.5	20 ± 2.5	7.1	7.9	6.5
2	1	42.8	40.1	38.3	42.3	40.9	38.2	−1.7	30.3	11.6
	2	39	39.4	38.2	35 ± 2.5	38 ± 2.5	36.4 ± 2.5	15.8	23.3	23.4

**Table 5:** Gains at 1600, 2000, and 2500 Hz with a frequency sweep from 1 kHz to 10 kHz at 65 dB SPL in the various configurations. Question marks (“?”) indicate that gain values could not be determined due to unstable high-frequency responses and measurement limitations (see Section 3.3).

Fit	Brand	HA + RM Output (Input 65 dB SPL)			HA + RM Output (Input 65 dB SPL)			HA + RM Output (Input 65 dB SPL)		
		High Frequency Gain (dB)			High Frequency Gain (dB)			High Frequency Gain (dB)		
		1600 Hz	2000 Hz	2500 Hz	1600 Hz	2000 Hz	2500 Hz	1600 Hz	2000 Hz	2500 Hz
1	1	15	21.9	24	?	?	?	4.4	29.1	22.4
	2	12.9	19	22.5	?	?	?	−8.4	18	16.5
2	1	36.5	41.6	41	?	?	?	28	45.1	38.4
	2	35.8	39.9	38.2	?	?	?	7.6	34.4	34.5

## Data Availability

The data supporting this study’s findings are available from the corresponding author upon reasonable request.

## Abbreviations

The following abbreviations are used in this manuscript:

RM	Remote Microphone
HA	Hearing Aid
CI	Cochlear Implant
SNR	Signal-to-Noise Ratio
FHSS	Frequency-Hopping Spread Spectrum
BTE	Behind-The-Ear
FIT1	First Fitting
CR	Compression Ratio
HTL	Hearing Threshold Level
UCL	Uncomfortable Loudness
FIT2	Second Fitting
AAA	American Academy of Audiology
ISTS	International Speech Test Signal
